# First Outbreak of African Swine Fever in Wild Boar in Nepal

**DOI:** 10.1002/vms3.70067

**Published:** 2024-10-09

**Authors:** Sameer Thakur, Chandrakala Rana, Naresh Prasad Joshi, Lokendra Neupane, Tirtha Raj Pokhrel, Avash Shrestha, Deepak Subedi

**Affiliations:** ^1^ Animal Service Department Dhangadhi Sub‐Metropolitan City Office Dhangadhi Nepal; ^2^ Department of National Parks and Wildlife Conservation Parsa National Park Office, Aadhabhar Bara Nepal; ^3^ Department of Livestock Services Veterinary Laboratory‐Dhangadhi Dhangadhi Nepal; ^4^ Department of National Parks and Wildlife Conservation Shuklaphanta National Park Office, Bhimdatta Kanchanpur Nepal; ^5^ Department of Poultry Science University of Georgia Athens Georgia USA

**Keywords:** African swine fever, economy, Nepal, pigs, wild boar

## Abstract

This case report documents the first confirmed outbreak of African swine fever (ASF) in a wild boar (*Sus scrofa*) in Nepal. The case was identified in a wild boar carcass found in Shuklaphanta National Park in February 2023. Post‐mortem findings, including splenomegaly, haemorrhagic lymph nodes and congested organs, were suggestive of ASF, which was confirmed by real‐time PCR. Epidemiological evidence indicates potential transmission from nearby ASF‐infected domestic pigs. This case underscores the critical need for rigorous biosecurity protocols, comprehensive surveillance and targeted wildlife management strategies to prevent further transmission between domestic pig and wild boar populations.

## Graphical abstract

We report the first confirmed African swine fever (ASF) outbreak in a wild boar (*Sus scrofa*) in Nepal, identified in Shuklaphanta National Park in February 2023. Post‐mortem examination revealed splenomegaly and haemorrhagic lymph nodes, and real‐time PCR confirmed ASF. Likely transmission from nearby infected domestic pigs highlights the urgent need for enhanced biosecurity, surveillance and wildlife management to prevent further spread between wild boar and domestic pigs.

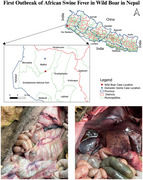

## 1 Introduction

African swine fever (ASF) is a highly contagious and fatal infectious disease affecting domestic swine and wild boar, with a mortality rate reaching up to 100% (Ruedas‐Torres, Thi to Nga, and Salguero 2024). The disease is caused by the ASF virus (ASFV), a large double‐stranded DNA virus belonging to the genus *Asfivirus* of the Asfarviridae family (Cho et al. 2023). Due to its significant socio‐economic impact and potential to spread across borders, ASF has been listed as a notifiable disease by the World Organisation for Animal Health (WOAH) (Penrith et al. 2024).

Discovered in Kenya in 1921, ASFV has since expanded its reach from Africa to other parts of the world, leading to substantial economic impacts on the pork industry. The current outbreak of ASF originated in Georgia in the Caucasus in 2007 and has since spread significantly across Africa, Europe and Asia in recent years (Dixon et al. 2020). The first outbreak of ASF in Asia began in domesticated pigs in China in 2018 and rapidly spread to East and Southeast Asian countries in 2019, including Mongolia, Vietnam, Cambodia, Hong Kong, Laos, Timor‐Leste, North Korea, Myanmar, the Philippines and Indonesia. Subsequently, the disease was reported in India in 2020, Malaysia, Bhutan and Thailand in 2021 as well as Singapore in 2023 (Ito et al. 2023). Nepal reported its first outbreak of ASF in March 2022 in domestic pigs aged 3–5 months in Kageshwori Manohara Municipality, Kathmandu, resulting in the death of 177 pigs (Subedi, Subedi, and Karki 2022; World Animal Health Information System [WAHIS] 2024). Since then, the disease has spread to 20 out of 77 districts across all 7 provinces of Nepal, leading to 24,508 susceptible domestic pigs, with 17,465 cases and 16,488 deaths as of 31 January 2024 (WAHIS 2024).

## 2 Case Description

**FIGURE 1:**
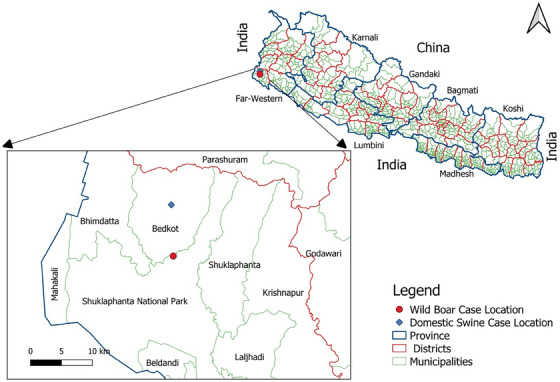
Map of Nepal showing the location of African swine fever case in wild boar in Shuklaphanta National Park and domestic pigs in Bedkot municipality, Kanchanpur District.

**FIGURE 2:**
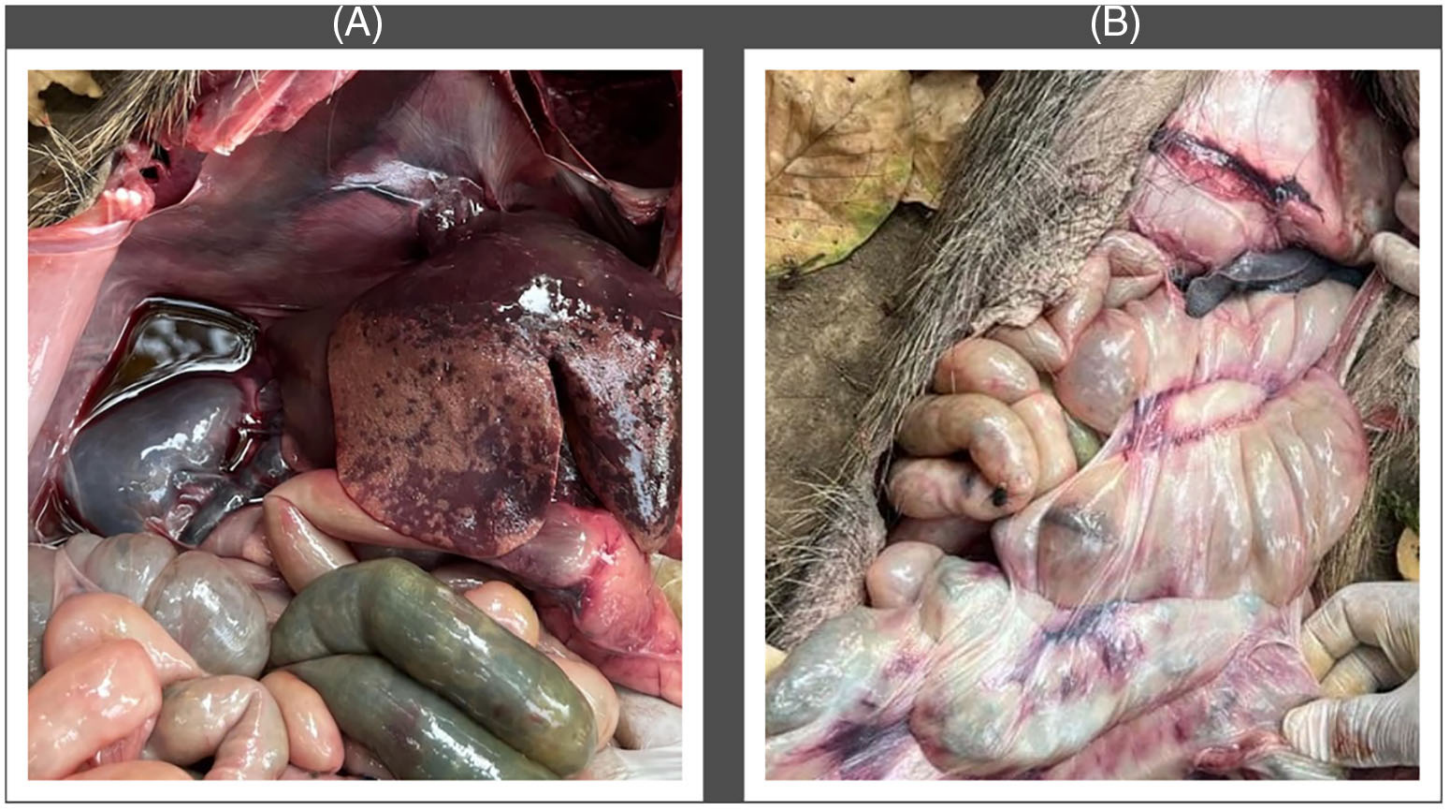
Post‐mortem finding of the wild boar, which was dead found in Shuklaphanta National Park, Kanchanpur, Nepal, due to African swine fever: (A) congested liver with multiple irregular dark patches and accumulation of moderate reddish fluid in the abdominal cavity; (B) haemorrhage and congestion in intestine and intestinal mesentery and enlarged spleen.

On 28 February 28 2023, a wild boar (*Sus scrofa*) approximately 7 months old was found dead in Shuklaphanta National Park (ShNP), Kanchanpur. ShNP (area 305 km^2^, buffer zone 243.5 km^2^) is one of the 12 national parks in Nepal, located in the Terai belt of the Far Western region of Nepal (28°45′–28°57′ N to 80°07′–80°21′ E). Out of 53 mammal species found in ShNP, wild boar is one of the major species (Pant et al. 2023), with an estimated population of approximately 1500. A carcass of an approximately 7‐month‐old female wild boar was reported dead on 28 February 2023, by the patrol team of Shree Rudradhwoj Battalion about 100 m north of Badhani Kheda post, ShNP, Majhgaun (Figure 1). The wild boar was found dead in left lateral recumbency with no external injury except for bleeding from the nose, and the body was slightly bloated. The incident was reported to the Veterinary Laboratory in Dhangadhi, Kailali, for further examination and investigation. The post‐mortem examination showed a slightly enlarged, dark‐coloured spleen; an enlarged, congested liver with multiple irregular dark patches; enlarged, haemorrhagic lymph nodes; and haemorrhage and congestion in the intestine and mesentery (Figure 2). Skin lesions were not observed in the carcass, as these are less common in wild boar than in domestic pigs (Sauter‐Louis et al. 2021). Additionally, there was an accumulation of moderate reddish fluid in the abdominal cavity. The post‐mortem findings pointed towards ASF as the cause of death. A large number of adult roundworms (*Trichuris* spp.) were found in the intestine along with nodules on the intestinal wall. Samples from the lungs, liver and mesenteric lymph nodes were collected for confirmatory diagnosis. The samples were transported to the Central Veterinary Laboratory (CVL), Kathmandu, maintaining the cold chain. At the CVL, a real‐time polymerase chain reaction (real‐time PCR) test confirmed the samples to be positive for ASF on 3 March 2023. This confirmed the first outbreak of ASF in wild boar in Nepal.

The ASF outbreak is primarily influenced by the close vicinity of ASF‐infected wild boar and domestic pig holdings (Boklund et al. 2020). Through epidemiological investigation, it seems likely that the disease was introduced through infected domestic pigs. The direct distance from the nearest detected ASF case on the domestic pig farm in Bedkot‐04, Kanchanpur, which was confirmed on 24 January 2023 (WAHIS 2024), to the case area of ShNP is 8.3 km. The case area is 1.5 km from the Mahendra (East–West) Highway, and there is no fencing in the case area of ShNP, leading to the free movement of humans and animals. The epidemiological cycle of ASFV in wild boars is characterized by a combination of local, endemic persistence with a simultaneous steady geographical spread to neighbouring disease‐free areas (European Food Safety Authority [EFSA] 2020). In the sylvatic cycle, the virus's survival in carcasses plays a vital role as it outlives its host. It can remain infectious at 4°C for over a year in blood, for several months in boned meat and for years in frozen carcasses (Fischer et al. 2020).

The outbreak of ASF in ShNP marks a significant event, highlighting the vulnerability of both wild and domestic pig populations to ASF. The confirmation of ASF in a wild boar within the park indicates a potential spillover of the virus from domestic swine populations. This incident underscores the complex interactions between wildlife and human activities, particularly in regions where agriculture and livestock farming are prevalent (Pant et al. 2023). Given the high mortality rates associated with ASF and its rapid spread across various regions, there is an urgent need for concerted efforts to prevent further transmission and mitigate its socio‐economic impacts. Immediate localization, safe removal and destruction of carcasses, along with decontamination of the environment, are crucial measures for the effective control of ASFV transmission in wild boars (Brown and Bevins 2018). Educating local communities about ASF and preventive measures will help reduce risky behaviours that contribute to the virus's spread (Acharya and Wilson 2020). Maintaining fences, controlling the wild boar population and monitoring as well as sampling wild boars in and around ShNP are advisable to prevent the further spread of ASF. It is crucial to implement enhanced surveillance programmes for both domestic pigs and wild boar populations, facilitating early detection and response to new cases. Strengthening biosecurity measures is essential to prevent transmission between domestic pigs and wildlife, including controlling pig movement and ensuring proper carcass disposal (Subedi et al. 2021). Conducting epidemiological studies will aid in understanding transmission pathways and developing targeted interventions. Collaboration among veterinary services, wildlife authorities and local governments is necessary for a coordinated response. Finally, wildlife management strategies should be developed to minimize contact between wild boars and domestic pigs, reducing the potential for cross‐species transmission.

## Author Contributions

Sameer Thakur, Chandrakala Rana, and Deepak Subedi performed conceptualization, data collection and writing of the original draft. Tirtha Raj Pokhrel and Lokendra Neupane conducted the post‐mortem of the dead wild boar and handled sample collection and dispatch for laboratory diagnosis. Naresh Prasad Joshi and Avash Shrestha contributed to conceptualization and data collection. Sameer Thakur and Deepak Subedi reviewed and finalized the manuscript. All authors have read and approved the final manuscript.

## Ethics Statement

The authors have nothing to report.

## Consent

All the authors have consented to the publication of the manuscript.

## Conflicts of Interest

The authors declare no conflicts of interest.

### Peer Review

The peer review history for this article is available at https://www.webofscience.com/api/gateway/wos/peer‐review/10.1002/vms3.70067.

## Data Availability

Data for this manuscript was available from Shuklaphanta National Park Office in Majhgaun and Veterinary Laboratory in Dhangadhi. There are no additional data beyond what is presented in the manuscript.
